# Evidence from Raman Spectroscopy of a Putative Link Between Inherent Bone Matrix Chemistry and Degenerative Joint Disease

**DOI:** 10.1002/art.38360

**Published:** 2014-04-28

**Authors:** Jemma G Kerns, Panagiotis D Gikas, Kevin Buckley, Adam Shepperd, Helen L Birch, Ian McCarthy, Jonathan Miles, Timothy W R Briggs, Richard Keen, Anthony W Parker, Pavel Matousek, Allen E Goodship

**Affiliations:** 1 Institute of Orthopaedics and Musculoskeletal Science, University College London, and Royal National Orthopaedic HospitalStanmore, UK; 2 Central Laser Facility, Science and Technology Facilities Council Rutherford Appleton LaboratoryDidcot, UK; 3 Royal National Orthopaedic HospitalStanmore, UK

## Abstract

**Objective:**

Osteoarthritis (OA) is a common debilitating disease that results in degeneration of cartilage and bone in the synovial joints. Subtle changes in the molecular structure of the subchondral bone matrix occur and may be associated with cartilage changes. The aim of this study was to explore whether the abnormal molecular changes observed in the matrix of OA subchondral bone can be identified with Raman spectroscopy.

**Methods:**

Tibial plateaus from patients undergoing total knee replacement for OA (n = 10) were compared with healthy joints from patients undergoing leg amputation (n = 5; sex- and laterality-matched) and with non-OA cadaveric knee specimens (n = 5; age-matched). The samples were analyzed with Raman spectroscopy, peripheral quantitative computed tomography, and chemical analysis to compare changes in defined load-bearing sites in both the medial and lateral compartments.

**Results:**

OA subchondral bone matrix changes were detected by Raman spectroscopy. Within each cohort, there was no spectral difference in bone matrix chemistry between the medial and lateral compartments, whereas a significant spectral difference (*P* < 0.001) was observed between the non-OA and OA specimens. Type I collagen chain ratios were normal in the non-OA specimens but were significantly elevated in the OA specimens.

**Conclusion:**

In comparing the results of Raman spectroscopy with those obtained by other standard techniques, these findings show, for the first time, that subchondral bone changes, or inherent differences, exist in both the medial and lateral (beneath intact cartilage) compartments of OA knees. The development of Raman spectroscopy as a screening tool, based on molecular-specific modifications in bone, would facilitate the identification of clinical disease, including early molecular changes.

Synovial joints allow movement, consist of multiple tissues and structures, and are considered to be skeletal organs. Osteoarthritis (OA) is a condition of multifactorial organ failure in which pathologic changes in the cartilage, bone, synovium, and other periarticular soft tissues interact (1). This structural failure causes inhibited function of the joint and, combined with chronic pain, results in debilitation and reduced quality of life.

Indications of disease progression include destruction of the cartilage in combination with abnormal thickening of the subchondral bone and gross deformity of the affected joint (2). The quality of bone, as a material, may have been underestimated in the understanding of the etiology and progression of disease (3). The interaction between cartilage and subchondral bone and its role in the pathogenesis of OA require further examination, and may provide a mechanical basis for the cartilage degradation process (4,5). It is possible that not one single tissue is responsible (6). Clarification as to whether specific biomolecular changes occur in the onset of OA (7,8) is needed.

To date, relatively little is known about the relationship between bone (material), structural competence, and the mechanobiologic interaction of bone and cartilage in the etiology of OA, particularly in the initiation and early stages of the disease. Two theories have been proposed. One theory postulates that OA is the result of altered impact mechanical loading, which induces bone adaptation with subchondral bone thickening, and leads to a stiffer structure (4). This new stiffer structure is less effective at absorbing shock, and therefore the distribution of force through the affected joint changes, leading to site-specific cartilage destruction. The “chemical” nature or quality of the newly synthesized bone as a material may be indistinguishable from the original bone tissue.

Another theory postulates that an alteration in bone matrix chemistry occurs as part of the disease progression. This hypothesis is supported by studies of OA bone, which show that the tropocollagen molecules comprise 3 α1 chains (rather than 2 α1 chains and 1 α2 chain) (9–12). Bone with homotrimeric collagen has a lower modulus (4.3 GPa) than that with heterotrimeric collagen (4.5 GPa), thus being less able to support the overlying cartilage without a compensatory increase in structural thickness (9,13). In addition, homotrimers have higher water content (14). In this setting, subchondral sclerosis or structural thickening could be a compensatory attempt to provide support for cartilage in the presence of abnormal bone matrix.

Conventional technologies, e.g., radiography, are used to assess the extent of damage in OA joints, particularly to identify joint space narrowing and thickened subchondral bone. Vibrational spectroscopic techniques, such as infrared or Raman spectroscopy, may also be applied. The main benefit of spectroscopic techniques over conventional techniques is the capability to detect the organic and inorganic phases of bone, effectively acquiring an overall biochemical signature, without destructive sample preparation (15).

Raman spectroscopy has been used to assess bone and pathologic changes in the tissue (16,17). Spectral analysis has shown that the hydroxyapatite:collagen ratio (mineral volume fraction), carbonate apatite:hydroxyapatite ratio (carbonate substitution), and amide III (protein conformation) are altered in subchondral bone from the hip joints of patients with OA (18), all of which are indicative of alterations in bone composition and the collagen secondary structure. In these studies, the nearby trabecular bone was not similarly affected, and analysis of samples from the most weight-bearing and least weight-bearing sites did not alter the outcome (18). The increased levels of homotrimeric collagen in the subchondral bone from OA femoral heads (8) may result in the alterations in the collagen secondary structure, as has been observed using Raman spectroscopy (18).

Raman spectroscopy is a technique that measures and quantifies the energetic changes in light (typically generated by a laser) scattered from materials. When light scatters from a sample, energy may be lost (or gained) by some photons and a shift in wavelength is observed to the red (energy loss) or the blue (energy gained) region. The shifts in wavelength of the photons are dependent on the chemicals within the material; therefore, Raman spectroscopy gives a chemical “fingerprint,” which, when analyzed, identifies the components present. The energy of the shifted light is plotted as a spectrum of the intensity of scattered light (y-axis) against the wave numbers (x-axis) (1 cm^−1^ = 1 × 10^7^/ wavelength in nm). Spectral bands for molecular functional groups associated with mineralized tissue include phosphate, carbonate, amides I and III (indicators of secondary protein conformation), proline, hydroxyproline, and lipids (19–21). The heights/areas of the bands can be compared, e.g., the mineral:collagen ratio, thus providing information on the organic and inorganic phases of bone and the degree of mineralization of a given specimen. Multivariate analyses of the Raman spectra can discern subtle differences that cannot be identified by eye. For example, principal components analysis (PCA) may be applied to analyze the spread of data and identify segregation of the spectra (22,23).

In this study, we explored the hypothesis that changes in bone matrix chemistry in the subchondral bone of the tibial plateau of patients with knee OA can be detected by Raman spectroscopy. Furthermore, the spectral distinction between OA and non-OA may be identified as changes in the organic phase (amide bands), which thereby result in a change in the mineral:collagen ratio. The load-bearing regions of both the medial (grossly affected) and lateral (not visually damaged at a macroscopic level) compartments of human tibial plateaus were probed independently, and samples were sex-matched (with a subset age-matched) to non-OA joints. The findings from Raman spectroscopy (overall biochemical signature) along with the findings obtained by peripheral quantitative computed tomography (pQCT) (mineral component) and the α-chain ratio (organic component) were assessed for correlations.

## PATIENTS AND METHODS

### Samples

**Figure 1:**
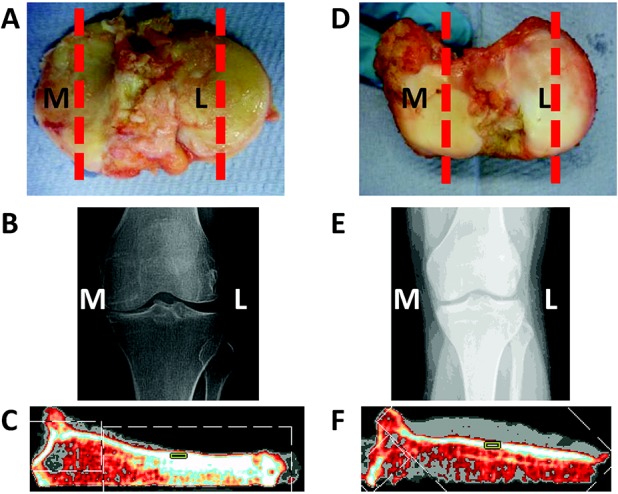
Assessment of the tibial plateau from a patient with osteoarthritis (OA) compared to that from a non-OA control. Postoperative photographs (**A** and **D**) and preoperative radiographs (**B** and **E**) of the medial (**M**) and lateral (**L**) tibial plateaus from a representative patient with OA (**A** and **B**) and a non-OA control (**D** and **E**) are shown. The medial tibial plateau (**C**) and lateral tibial plateau (**F**) from a patient with OA were assessed by peripheral quantitative computed tomography (pQCT). In the pQCT images, the region of interest is demarcated as a green box. The red broken lines in **A** and **D** indicate the plane from which the pQCT measurements were obtained.

Human tibial plateaus were acquired from patients with knee OA (n = 10) following the patients' provision of informed consent and ethics approval from the Royal National Orthopaedic Hospital, UK (ethics approval no. 08/H0304/78). The tibial plateaus were obtained from patients undergoing total knee replacement for established grade IV (Outerbridge classification) medial compartment OA (radiographic and macroscopic diagnosis). Control specimens were collected from non-OA patients undergoing various operations (due to different conditions of the proximal femur, with no evidence of tibial involvement, no OA of the knee joint, and without changes in clinically assessed load-bearing) requiring removal of the leg (n = 5). In addition, control samples were obtained from non-OA cadaveric specimens (n = 5, from 3 age-matched subjects) at Vesalius Clinical Training Centre, University of Bristol (ethics approval no. 08/H0724/34). The patients with OA (mean ± SD age 68 ± 15 years) and the non-OA subjects (mean ± SD age 75 ± 15 years for the cadaveric specimens and 30 ± 18 years for the amputees) were matched for sex and laterality. All non-OA specimens were examined by an orthopedic surgeon, and no visual appearance of OA was found, either macroscopically or radiographically. Figure 1A shows a typical OA specimen, and Figure 1B is the corresponding radiograph with evidence of clear joint space narrowing and thickening of the medial subchondral bone. Figures 1D and E show a non-OA equivalent specimen.

Samples were frozen at −80°C within 2 hours of removal, for storage prior to analysis. Cadaveric specimens were kept in storage at −20°C at the Vesalius Clinical Training Centre within 48 hours of death, and subsequently transported frozen.

### Peripheral QCT

The medial and lateral compartments of the tibial plateaus were scanned using pQCT (XCT 3000; Stratec) to determine volumetric bone mineral density (vBMD) and thickness of the subchondral bone (Figures 1C and F). The x-ray beam width was 2 mm and the voxel size was fixed at 0.3 mm. Specimens were measured at 5-mm intervals across the tibial plateau. A measurement slice from the center of each compartment of the plateau (demarcated with the red broken lines in Figures 1A and D) was used to calculate the vBMD (in mg/cm^3^) of the subchondral bone; the regions of interest used were the same size and in the same location across all measurements (Figures 1C and F). Additionally, the thickness of the subchondral bone was measured (in mm).

### Raman microspectroscopy

Cylindrical cores (dimension ∼12 mm^2^ × height of plateau [range 10–20 mm]) were extracted from the tibial plateaus using an 11-gauge Jamshidi Crown bone marrow biopsy needle (Cardinal Health, France), with 2 samples obtained from each compartment, corresponding to the same sites analyzed by pQCT. The cores were turned on their side, and spectra were acquired directly from the subchondral bone (≤3 mm below the cartilage, if present) using an InVia Raman microspectrometer (Renishaw). This was equipped with an 830-nm laser, 300 mW at source. Calibration was performed every day on silicon, as silicon has a known Raman band (520.5 cm^−1^), and on polystyrene to measure bands in the same range as that of bone (wave numbers between 1000 cm^−1^ and 1030 cm^−1^). There was no difference across the values during the time of data collection. The spectra were acquired at a laser power of 2 mW for 1 second and 4 accumulations; 5 different spectra were acquired from the subchondral bone per core (total number of spectra = 400). The spatial resolution (at 50× objective) was 2 μm × 2 μm. There was no thermal heating or degradation of the samples at the laser powers utilized.

### Lipid removal

The removal of lipids was required because lipids are strong light scatterers on Raman microspectroscopy, having spectral peaks that overlie some of the spectral peaks from bone matrix. Following established bone preparation techniques (24,25), the cores were washed in 5 ml of acetone on a roller for 1 hour at 37°C with constant agitation. The acetone was removed and replaced with fresh acetone twice. The cores were then rinsed in distilled H_2_O to remove the acetone. The spectral measurements were then repeated with the same parameters. The effect of lipid removal with acetone was validated by comparing peak ratios, namely the ratios of phosphate to amide I and phosphate to carbonate, before and after lipid removal (details available from the corresponding author upon request).

### Polarization and orientation

The laser used generates polarized light, and certain materials/molecular bonds that are preferentially orientated with respect to the laser polarization can be more or less efficient at scattering light. Therefore, it was important to assess the influence of polarization and orientation on these specimens. All spectra, as described above, were acquired without the polarizer and at the same orientation (within 10°). Additional spectra were acquired with a polarizer in place, i.e., the laser was fully polarized, under the same parameters and from the same location (within 1 μm) every 45° from 0° to 360°. Spectra were then processed as described below, and the mineralization ratios were calculated for the ratio of phosphate (η_1_; 960 cm^−1^) to amide I and the ratio of phosphate (η_4_; 588 cm^−1^) to amide III; of note, the former measure, being a symmetric mode, is more sensitive to polarization (26) (details available from the corresponding author upon request).

### Type I collagen α-chain analysis

#### Sample preparation

Defatted cores of the subchondral bone were weighed and then decalcified in 10 ml 10% EDTA for 1 week on a roller at 4°C. The cores were washed with deionized water (to remove EDTA), and then reweighed and freeze-dried. Five milligrams from each sample was isolated for further processing. These cores were immersed in 1 ml 0.5*M* acetic acid with 25 μg pepsin (porcine gastric mucosa, 3,200–4,500 units/mg protein; Sigma) added at 0.5% weight/weight of 5 mg cores. Samples were left to digest with agitation for 2 days at 4°C. The supernatants were collected following centrifugation at 3,000 revolutions per minute for 30 minutes at 4°C, and freeze-dried overnight.

#### Sodium dodecyl sulfate–polyacrylamide gel electrophoresis (SDS-PAGE)

The samples were dissolved in 0.5 ml of SDS-PAGE sample buffer (125 m*M* Tris [pH 6.8], 2% SDS, 10% glycerol, 0.01% bromphenol blue). SDS-PAGE was performed using 7.5% polyacrylamide resolving gel matrix and 4.5% stacking gel in a Mini-Protean II apparatus (Bio-Rad, UK). After electrophoresis, the gels were stained with Coomassie blue (0.005%) to visualize protein, and then destained with 10% acetic acid and 20% methanol solution. A type I collagen standard prepared from equine skin and molecular weight markers were run alongside the samples. Following staining and destaining, a digital image of the gel was obtained, and ImageJ software was used to quantify the intensities of the α1- and α2-chain bands (details available from the corresponding author upon request). The α1:α2 chain ratio was calculated using a correction factor (1.16) to account for the smaller α2 polypeptide chain.

### Statistical analysis

**Figure 2:**
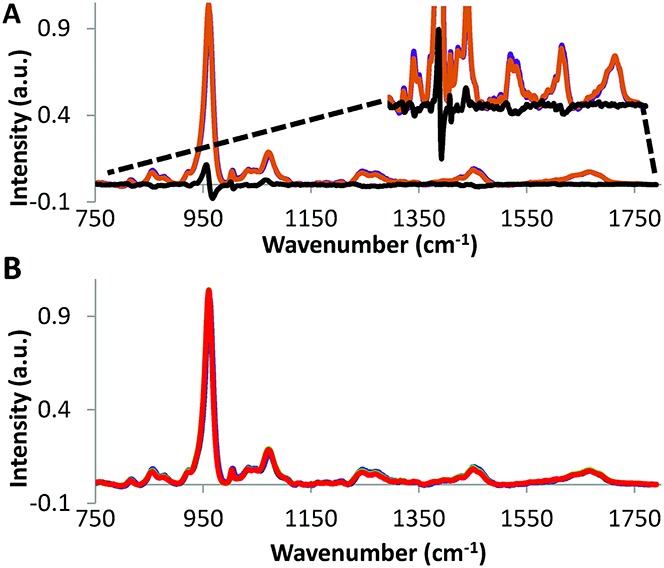
Average intensity of the Ramen spectra (wave numbers 750–1800 cm^−1^) in **A,** non-osteoarthritis (non-OA) (purple) versus OA (orange) tibial specimens (difference shown in black), and in **B,** non-OA medial (black) versus non-OA lateral (blue) compartments, and OA medial (green) versus OA lateral (red) compartments.

Spectra were baseline corrected (750–1800 cm^−1^) using a third-order polynomial (Matlab; The Mathworks), with values normalized to the phosphate peak (960 cm^−1^). For multivariate analyses, PCA (unsupervised) and PCA-linear discriminant analysis (PCA-LDA; supervised) were performed to facilitate the identification of segregation of the spectra, based on variance, by forming linear combinations of the wave numbers and ranking them in order of variance (Matlab 2012a; The Mathworks) (27,28). The Raman spectra in each cohort were averaged (Figure 2). Subjectively, there were few spectral differences between the OA and non-OA samples; however, subtle differences are difficult to identify by observing the average spectra. A 3-dimensional scatter plot of the resulting scores obtained from the multivariate analysis (PCA) allows for differences to be identified objectively, because the distance between the scores (expressed as points; each point represents a spectrum) on the plot is proportional to the spectral, and therefore the biochemical, similarities.

Univariate analysis was utilized for direct comparison of the bands of interest, i.e., the ratio of phosphate (960 cm^−1^) to amide I (1660 cm^−1^), the ratio of phosphate to proline (920 cm^−1^), the ratio of phosphate to carbonate (1070 cm^−1^), and the ratio of phosphate to hydroxyproline (885 cm^−1^ + 870 cm^−1^), which provides the relative amount of bioapatite to collagen (29). Peak heights were compared to determine the ratios. The α-chain ratios were analyzed using the Mann-Whitney U test, as a nonparametric equivalent to the independent-samples *t*-test (SPSS; IBM UK). One-way analysis of variance, followed by the Bonferroni post hoc test, was used to compare the density, thickness, and median spectral ratios across the multiple groups (Origin 8.6; OriginLab). Throughout the statistical testing, the number of subjects per cohort was noted.

## RESULTS

### Density and thickness of the subchondral bone

**Figure 3:**
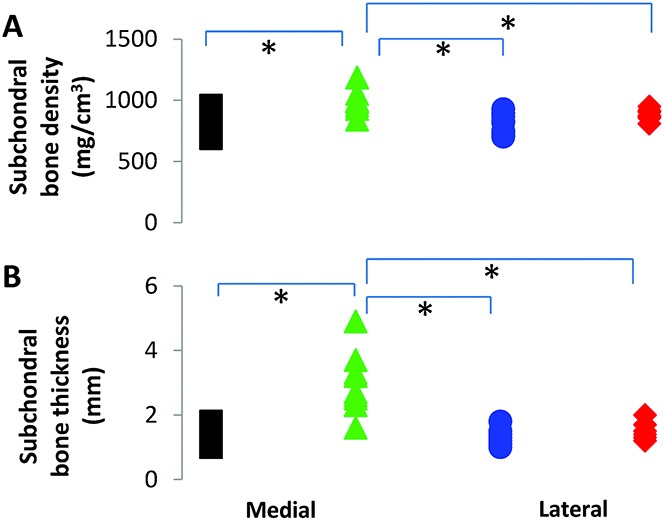
Results of peripheral quantitative computed tomography assessing **A,** the subchondral bone density and **B,** the subchondral bone thickness of each tibial compartment, comparing the medial (black squares) and lateral (blue circles) compartments of non-osteoarthritis (non-OA) tibial plateaus and the medial (green triangles) and lateral (red diamonds) compartments of OA tibial plateaus. * = *P* < 0.05.

The density of the subchondral bone was significantly higher across the OA tibial plateaus (mean ± SD 950 ± 100 mg/cm^3^) compared to the non-OA samples (mean ± SD 820 ± 90 mg/cm^3^; *P* < 0.001) (Figure 3A). In comparing the tibial compartments, the medial subchondral bone had a significantly higher structural density than the lateral subchondral bone (mean ± SD 940 ± 94 mg/cm^3^ versus 884 ± 25 mg/cm^3^; *P* = 0.005) in all OA specimens, whereas only 3 of the 10 non-OA specimens showed a difference in density, albeit not significant (*P* = 0.2), between compartments. Moreover, the OA medial compartments were significantly denser than the non-OA medial compartments (*P* = 0.003) (Figure 3A).

With regard to subchondral bone thickness, the medial compartment of the OA specimens was significantly thicker than the lateral compartment, and was significantly thicker in OA specimens compared to non-OA specimens (Figure 3B). The thickness of the non-OA medial and lateral compartments was comparable (mean ± SD 1.3 ± 0.2 mm).

### Raman spectral signatures

#### Non-OA versus OA

**Figure 4:**
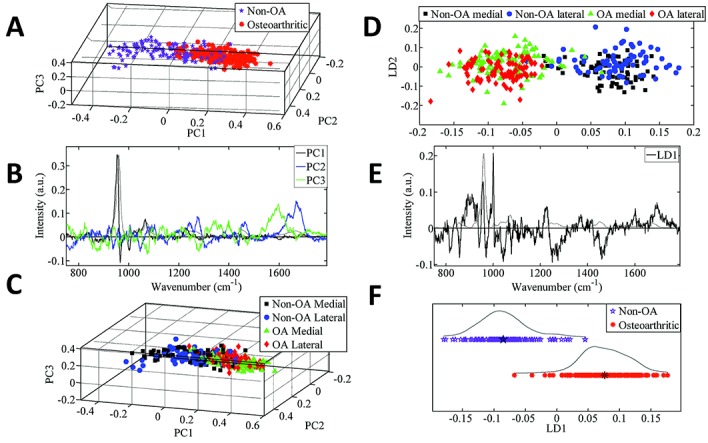
Principal components analysis (PCA) of the Ramen spectra. **A–C,** Plot of PCA scores for the non-osteoarthritis (non-OA) tibial specimens compared to the OA tibial specimens **(A)**, the corresponding PCA loadings plot **(B)**, and the color-coded plot of PCA scores in the non-OA and OA medial and lateral compartments (color-coded to enable identification of the medial and lateral spectra) **(C)**. **D–F,** Plot of PCA–linear discriminant analysis (PCA-LDA) scores for the non-OA and OA medial and lateral compartments **(D)**, the corresponding PCA-LDA loadings plot **(E)**, and the plot of PCA-LDA scores for the non-OA tibial specimens compared to the OA tibial specimens (**F**). The data in **F** are plotted along the x-axis, and split along the y-axis only for ease of visualization. The loadings plots **(B** and **E)** show the axes (linear combination of the variables) for the PCA-LDA and LDA analyses, respectively. In loadings plots, the larger the peak, the more influence it has on any separation in the scores plotted along a particular axis.

PCA revealed a large separation of spectra (confidence interval 0.95) between the non-OA and OA specimens (Figure 4A). However, there was no difference between the medial and lateral compartments within each cohort (Figure 4C).

The spectral differences resolved from PCA can be identified by interpreting Figure 4B. The first axis (PC1) shows that the phosphate band (954 cm^−1^ and 966 cm^−1^) was different between the cohorts. The second axis (PC2) reveals that the second largest difference was in amide I (1668 cm^−1^ and 1685 cm^−1^) and the phosphate shoulder (941 cm^−1^). Finally, the third axis (PC3) shows that the next difference was related to broad differences across ∼1597 cm^−1^.

Univariate analysis of the Raman spectral peaks (Table 1) revealed statistically significant differences between the cohorts for the phosphate:amide I ratio (*P* = 0.04) and the bioapatite:collagen ratio (*P* = 0.04), in the medial and lateral compartments combined.

**Table 1:** Results of Raman spectral and biochemical analyses*

	Raman spectral analysis			
		
	Phosphate:amide I	Carbonate:phosphate	Bioapatite:collagen	Biochemical analysis, α1:α2 chain ratio
Non-OA				
Medial compartment	13.12 (4.47)	0.17 (0.02)	8.5 (1.92)	2.0 (1.03–3.15):1.0
Lateral compartment	13.45 (3.36)	0.17 (0.01)	8.99 (2.98)	2.0 (1.44–3.62):1.0
*P* for comparison	1	1	1	0.5
OA				
Medial compartment	13.96 (2.98)	0.18 (0.02)	9.90 (2.11)	3.0 (1.58–8.08):1.0
Lateral compartment	14.70 (3.56)	0.18 (0.01)	10.7 (2.42)	2.3 (1.63–9.90):1.0
*P* for comparison	1	1	0.3	0.4
Medial + lateral compartments				
Non-OA	13.28 (3.86)	0.17 (0.02)	8.75 (2.38)	2.0 (1.03–3.62):1.0
OA	14.33 (3.47)	0.18 (0.02)	10.3 (2.27)	2.6 (1.58–9.90):1.0
*P* for comparison	0.04†	0.1	0.04†	0.2
Medial compartment				
*P*, non-OA vs. OA	0.4	0.3	0.2	0.03†

* Results of the Raman spectral analysis are the median ratio (interquartile range) determined by univariate analysis for bone mineralization (ratio of phosphate to amide I), bone turnover (ratio of carbonate to phosphate), and the bioapatite:collagen ratio in osteoarthritis (OA) and non-OA tibial plateau specimens. Results of the biochemical analysis are the ratio of type I collagen α1 chain, expressed as the median (range), to α2 chain for each cohort.

† *P* value is statistically significant at the 95% confidence level.

#### Medial versus lateral compartments

PCA-LDA was used to separate the 4 groups, comprising comparisons of the non-OA medial versus non-OA lateral compartments, and OA medial versus OA lateral compartments. The findings, shown in Figure 4D, confirm that there were differences between the non-OA and OA specimens, and that there were no differences between compartments within each cohort. The chemical components that showed the most differences in intensity between the non-OA and OA specimens, regardless of compartment (Figure 4B), were hydroxyproline (858 cm^−1^), C–C collagen backbone (941 cm^−1^), and phosphate (956 cm^−1^ and 966 cm^−1^).

#### Medial non-OA versus medial OA, and lateral non-OA versus lateral OA

**Figure 5:**
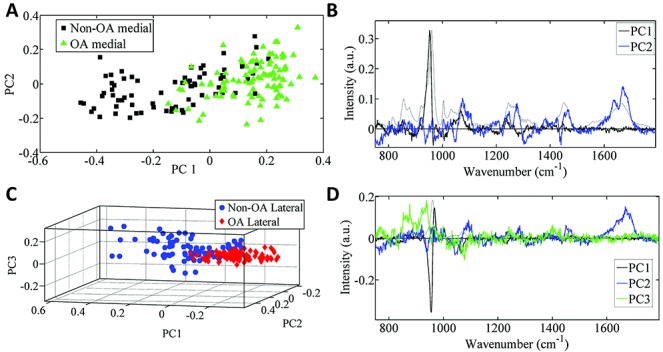
Principal components analysis (PCA) showing the plot of PCA scores for the non-osteoarthritis (non-OA) and OA medial compartment **(A)** and the corresponding loadings plot **(B)**, as well as the plot of PCA scores for the non-OA and OA lateral compartment **(C)** and the corresponding loadings plot **(D)**.

Analysis of the non-OA medial compartment compared to the OA medial compartment (Figures 5A and B) revealed a spectral separation that could be attributed to differences in the intensity of phosphate (944 cm^−1^ and 953 cm^−1^), amide III (1275 cm^−1^), and a broad region across amide I (1650 cm^−1^). Analysis of the lateral compartment in non-OA specimens compared to OA specimens (Figures 5C and D) indicated that there was a biochemical difference, with more intracategory variance, in the non-OA specimens. The spectral bands contributing to the differences were the bands at 850 cm^−1^ and 910 cm^−1^, and the phosphate bands (963 cm^−1^ and 956 cm^−1^).

#### Polarization

Analyses of the results from the polarization/orientation tests showed that the Raman spectra were sensitive to sample orientation, but the variations were smaller than the differences between the samples (details available from the corresponding author upon request). Therefore, we can conclude that polarization had no effect on our findings.

### Biochemical findings

The OA specimens had a significantly higher α1:α2 chain ratio compared to the non-OA specimens (*P* = 0.03) (Table 1). The largest α1:α2 chain ratio in the non-OA specimens was 3.62:1.0 (lowest ratio 1.03:1.0; mean ratio 2.0:1.0). In the OA specimens, the largest α1:α2 chain ratio was 9.90:1.0 (lowest ratio 1.58:1.0; mean ratio 2.6:1.0).

## DISCUSSION

Results from the pQCT, Raman spectral, and collagen analyses all showed significant cohort-specific differences between the OA and non-OA samples. The results support our hypothesis that bone matrix changes found in OA specimens can be detected with Raman spectroscopy. An unexpected new finding is that this spectral difference between OA and non-OA tissue was discernible in both the lateral and medial compartments. The OA medial compartment was expected to be different, as all OA specimens were confirmed to have grade IV OA (30) in the medial compartment. The previously reported enhanced levels of homotrimeric type I collagen in OA bone were confirmed by our findings in the grossly affected OA medial compartment. However, elevated levels of homotrimeric collagen and associated spectral differences were also found in the grossly normal lateral compartment of the OA specimens, indicating that the whole joint is affected.

Damage and repair may enhance tissue turnover in individuals with OA, e.g., during weight-bearing. Previous studies have suggested that the high tissue turnover rate and proliferation lead to thickening of the subchondral bone (31). Differences in the lateral compartment suggest that underlying biochemical changes are taking place, leading to dissemination of gross pathologic alterations across the joint. Although the lateral compartment is not showing macroscopically visible/symptomatic changes toward OA, it is exhibiting small changes, suggestive of early signs of OA, or developing inherent differences in the bone matrix.

OA of the knee is a painful condition that changes the loading of the limb, and therefore mechanobiologic factors may be a part of the changes observed. Furthermore, the OA cohort could be exhibiting an inherent difference in bone matrix chemistry, with associated material properties, which would predispose individuals to mechanobiologic joint degeneration. This theory is speculative, but it is known that there can be different responses of bone to increased and decreased mechanical loading (32), and that the material properties of bone across different (nonmutant) strains of a single species (of mice) can vary by up to 27% (33). The current findings may contribute evidence to suggest that individual variation is more complex than previously known, and therefore the findings warrant further investigation to determine whether they are inherent genetic differences or preclinical manifestations of disease. Confirmation of either finding would be significant, particularly in providing opportunities for early diagnosis.

Subchondral bone thickness from a healthy joint is known to be in the average range of 1 mm (±SD 0.28) to 1.2 mm (±SD 0.41) for both compartments (34). Consistent with this, the mean ± SD measurement of subchondral bone thickness in this study, in each of the non-OA compartments and the OA lateral compartments, was 1.2 ± 0.23 mm. Comparatively, subchondral bone in the OA medial compartment was significantly thicker (3.0 ± 0.8 mm), which is consistent with previous observations in which OA subchondral bone was noted to be thicker (6). Interestingly, the density of the OA subchondral bone was increased in the lateral and medial compartments.

The differences observed between non-OA and OA specimens could be attributed to the spectral signatures associated with phosphate, amide I, and phenylalanine tissue components. This suggests that there are changes in collagen that may affect mineralization of the subchondral bone. The spectral, and therefore biochemical, difference between OA and non-OA subchondral bone should be explored further to identify potential therapeutic targets for new pharmacologic treatments of early-stage OA and preclinical OA. Early-stage treatment could result in a delay or reduction in joint replacements.

The medial compartments in the OA and non-OA specimens could be distinguished by differences in the low–wave number shoulder of the phosphate peak and in the amide I peak. However, differences in the lateral compartments between OA and non-OA specimens were associated with hydroxyproline/proline, amide I, and centroid of the phosphate peak, supporting the theory that there are collagen changes in the bone. The hydroxylation of proline is an important contributor to the strength of collagen, as it forms hydrogen bonds and water bridges, which stabilize the triple helix (35,36). The distinguishable changes across the 2 compartments could identify a progression at the late stage of OA (medial) compared to the early stage of OA (lateral).

The higher mineralization ratio (phosphate:amide I) in OA compared to non-OA specimens was consistent with the pQCT density measurements, and suggests that a change/difference in the bone matrix composition precedes subchondral bone thickening. These findings support the theory that a material change to subchondral bone, rather than just an increase in volume, occurs. The second band ratio calculation that supports this finding is the bioapatite:collagen quantification, which shows that OA subchondral bone, as compared to non-OA specimens, has more mineral than collagen. This demonstrates a definable difference between the OA and non-OA specimens that can be resolved by Raman spectroscopy.

Lipid removal was successful, as confirmed by a lack of lipid-specific peaks at 1300 cm^−1^ and 1750 cm^−1^(details available from the corresponding author upon request). This process was important, as lipids are strong Raman scatterers and their presence could mask the bone at the small spatial resolutions that were utilized. The polarization/orientation effects seen were smaller than the differences due to disease (results available from the corresponding author upon request). Furthermore, since all of the specimens were measured in the same orientation (within 10°), any impact from polarization effects would be minimized. A recent study of polarization effects on bone confirms the orientation-induced changes in the peaks. The bioapatite:collagen peak is the most reliable, as the proline and phosphate peaks are phase-matched peaks, i.e., they change in proportion to polarization/orientation (37). In addition, the validation study revealed that the OA specimens were more sensitive to polarization/orientation than the non-OA specimens.

Considering the potential of Raman spectroscopy as a diagnostic tool, the recently developed technique of spatially offset Raman spectroscopy (SORS) (38) allows the acquisition of spectra from up to 4 mm below the surface, e.g., bone through skin, or through cartilage arthroscopically (39–43). As these approaches in a clinical setting would be minimally invasive and nondestructive, and would not involve ionizing radiation, they have the potential to be a powerful complementary technique to current technologies. SORS, as a noninvasive, nonionizing technique able to probe both the organic and inorganic phases of bone, has potential to be used as a diagnostic tool in vivo. The delivery of the laser and collection of the Raman photons further minimizes the effects of polarization/orientation associated with the laser, and the millimeter scale and depth measurement should reduce the contribution of lipids and probe a larger area of bone, thus minimizing the effect of heterogeneities within the bone structure.

Results of the biochemical analyses showed that there was less homotrimeric collagen (ratio range 1.6:1 to 9.9:1) than that previously reported (ratio range 4:1 to 17:1) (8). There may be several reasons for this. First, this study used bone from tibial plateaus, whereas the levels previously reported used femoral heads (8). Second, the area measured in the femoral heads was a minimum of 1 cm below the cartilage (mostly epiphyseal trabecular bone), and therefore unlikely to include subchondral bone, so there cannot be a direct comparison between our data and those previously reported (8).

Studies have shown that homotrimeric type I collagen molecules have an increased hydroxyproline content and increased denaturation temperature compared to heterotrimeric molecules (14). An increased molecular stability is further supported by the finding that resistance to cleavage by collagenase is increased due to less efficient unwinding at the cleavage site (44). The α1 chain is less hydrophobic than the α2 chain, which may result in increased water content of the fibril and, consequently, increased spacing between collagen molecules (14). The increased distance between collagen molecules has been implicated in the reduction in immature collagen crosslink levels and decreased mechanical strength observed in type I homotrimer *oim* mice (14). Furthermore, because mineralization occurs preferentially in hydrophilic environments, this may explain the increased mineralization levels of the OA bone. Molecular simulation studies have suggested that homotrimeric collagen molecules are more flexible and form kinks more freely than do heterotrimeric molecules, providing an alternative explanation for the increased spacing and decreased intermolecular crosslinking in homotrimeric collagen (45). In previous studies, the presence of homotrimeric collagen was associated with a higher rate of collagen turnover (11). However, in our study, we found no evidence of increased matrix turnover.

In summary, in the present study, differences were found between non-OA subchondral bone and the grossly affected medial compartment of OA subchondral bone. We were able to detect these changes using a nondestructive spectroscopic technique. Furthermore, these differences were comparable to that in the OA subchondral bone beneath macroscopically intact cartilage of the lateral compartment. There was a large significant difference between the profiles of the non-OA and OA specimens, which could be due to predisposition, increased turnover, or change in loading across the joint. The study results also indicated that the subchondral bone matrix chemistry across the whole joint was affected, possibly due to different stages through the progression of the disease, and thus the mineralization process was affected. This supports research previously reported in animal models (46,47).

Our findings thus indicate that subchondral bone changes, or inherent differences, exist in both the medial and lateral compartments of the OA tibial plateau. Furthermore, changes can be found beneath visibly unaffected cartilage of the lateral compartment, indicating, for the first time, that the joint as a whole is predisposed to develop OA. Subchondral bone therefore represents a key target for therapeutic strategies (mechanical or pharmaceutical). The detection of bone matrix chemistry variations, by Raman spectroscopy coupled with multivariate analysis, in subchondral bone would facilitate the identification of clinical disease, including early molecular changes. It is possible that these changes may be inherent to the individual, and therefore a better understanding of the changes would enable identification of those at risk of OA.
